# Autonomous learning of generative models with chemical reaction network ensembles

**DOI:** 10.1098/rsif.2024.0373

**Published:** 2025-01-22

**Authors:** William Poole, Thomas E. Ouldridge, Manoj Gopalkrishnan

**Affiliations:** ^1^ California Institute of Technology, Pasadena, CA, USA; ^2^ Imperial College London, London, UK; ^3^ Indian Institute of Technology Bombay, Mumbai, Maharashtra, India

**Keywords:** chemical reaction network, Boltzmann machine, molecular programming, generative models, gradient descent, feedback control

## Abstract

Can a micron-sized sack of interacting molecules autonomously learn an internal model of a complex and fluctuating environment? We draw insights from control theory, machine learning theory, chemical reaction network theory and statistical physics to develop a general architecture whereby a broad class of chemical systems can autonomously learn complex distributions. Our construction takes the form of a chemical implementation of machine learning’s optimization workhorse: gradient descent on the relative entropy cost function, which we demonstrate can be viewed as a form of integral feedback control. We show how this method can be applied to optimize any detailed balanced chemical reaction network and that the construction is capable of using hidden units to learn complex distributions.

## 1. Introduction

Living organisms demonstrate a remarkable ability to thrive in diverse conditions, survive perturbations and generally adapt to their environments [1]. In contrast, many synthetic *in vitro* and *in vivo* biochemical circuits require fine-tuning to operate well [2]. These observations are partially reconciled by the substantial experimental and theoretical evidence that biological circuitry has evolved to be inherently adaptive and robust [3,4].

The ideas of adaptation and robustness are central in biology and medicine, often discussed under the moniker of homeostasis [5]. The principle of homeostasis states that aspects of the internal environment of an organism (such as a cell) must be held relatively constant to maintain the vitality of the organism; in this sense, homeostasis is a dynamic process which enables life to thrive in diverse fluctuating environments [6]. Homeostasis has frequently been studied mathematically using tools from control theory that emphasize how feedback mechanisms are capable of dynamically adjusting internal variables of a system [7]. Robust perfect adaptation is a particularly well-studied kind of homeostasis by which an internal variable can be held at an exact value despite uncertainty and variability in the dynamics of the entire system [8]; it has been shown formally that for some classes of systems, a sufficient condition for robust perfect adaptation is integral feedback [9]. Furthermore, robust perfect adaptation has been studied and observed in many biological systems including bacterial chemotaxis, calcium homeostasis and others [9–11]. The internal model principle of control theory offers another valuable lens for understanding how biochemical systems may be capable of homeostasis. This principle states that for a system to adapt to a fluctuating environment, the system must have an internal model of the kinds of fluctuations present in that environment and how they affect the system [12–14].

Machine learning and neural networks, although originally developed for understanding intelligent behaviour in biological brains and for engineering analogous behaviour in electronic computers, have increasingly been applied as a theoretical framework for complex adaptive behaviour in non-neural physical systems [15] and, of particular relevance here, in single-cell organisms [16–18]. Beyond the relevance to biological systems, neural network architectures have inspired attempts to theoretically formulate and experimentally implement artificial chemical systems with similar computational abilities [19,20]. As a proof-of-principle example, theoretical chemical systems have been described that implement supervised learning of deterministic functions by backpropagation [21]. In the context of biological control and homeostasis, it has been suggested that learning architectures could be used by chemical systems to increase their homeostatic capacity via adaptive internal models [18].

As an alternative to deterministic function evaluation, generative machine learning architectures have emerged as a powerful framework for representing the distributions underlying complex systems [22]. These systems have the abilities to both represent diverse distributions and learn these representations from data [23]. The utility of generative models to living organisms has been recognized as biologically relevant in neuroscience. Theories such as predictive coding [24] and the free energy principle [25] posit that the brain is, at some level, a generative model making predictions about its environment and learning by comparing those predictions to sensory observations. Although some of these principles have begun to be explored in the context of molecular and developmental biology [26], it remains an open question to what extent these theories apply to molecular biology.

Chemical reaction networks (CRNs) provide a powerful formalism rooted in statistical physics to understand biomolecular systems [27]. CRNs have been used to study systems such as gene regulation, signalling and stochasticity in biology [28,29]. In the control context, CRNs have been used to study integral feedback in biochemical systems and to design synthetic biochemical control circuits [30]. Additionally, when studied as formal models of computation, CRNs have been proven to be Turing universal [31] and capable of implementing neural architectures both in theory [19,32] and in laboratory settings [33]. Importantly, CRNs have been proven to be capable of producing an arbitrary distribution [34], including Boltzmann machines, an important example of generative models [35,36]. These demonstrations indicate that CRNs have the potential to be powerful generative models; however, it is less clear how these generative models can adapt and learn from an external environment.

There is currently limited understanding of learning in stochastic CRNs that represent generative models. Within a CRN that generates a probabilistic choice based on a single probability, a technique based on operant conditioning effectively adapts the counts of ‘weight species’ to optimize a feedback signal [37]. We aim at two improvements over this scenario. First, we are interested in learning architectures suitable for classes of CRNs that are capable of representing arbitrarily complex distributions, and second, we wish to exploit the stochasticity that arises at equilibrium to emphasize the potential energy efficiency of generative models within chemical systems.

In prior related work, we examined how a class of chemical systems called detailed balanced CRNs (which will be formally defined in the §§2.3 and 2.4) can be viewed as a kind of generative model capable of probabilistic inference [38]. In this article, we extend these results to show how detailed balanced CRNs are capable of *learning* from a noisy environment when connected to a chemical module that implements the well-known machine learning optimization algorithm gradient descent [36], which we rigorously show is related to feedback control. We then use these results to design a fully autonomous chemical system that is capable of learning complex environmental distributions. Crucially, we show that this construction is capable of engaging hidden units (e.g. latent variables) to build an expressive internal model of the environmental distribution.

## 2. Background

The following sections provide overviews of the relevant background needed to understand the main results of this work. Additionally, we refer the reader to electronic supplementary material, appendix A for a succinct overview of the notation and conventions used in this work.

### 2.1. Learning

The *learning* in machine learning is a process by which a mathematical function representing some kind of computation is automatically optimized from data. In the context of this article, we are interested in a class of functions called graphical generative models, which can be sampled to produce probability distributions P(x∣λ) where x are state variables (typically vectors of real, integer or binary values) and λ are parameters (typically real valued), of the distribution [23].

The learning process can be formalized as optimizing a loss function L with an algorithm such as gradient descent to find parameters $\lambda^{*}$ from data sampled from the distribution ψ


(2.1) $$\begin{array}{lcr} & \lambda^{*}=\underset{\lambda }{\text{argmin}}\;L(\mathbb{P}(x|\lambda ),\psi ). & \end{array}$$

It is worth emphasizing that learning is a dynamic process in which the parameters λ are updated iteratively based upon samples drawn from the distribution ψ. Importantly, the underlying data generating distribution ψ is typically unknown; for example, it may be a complex stochastic process such as the biochemical reactions in a cell. Instead, samples drawn from ψ, (e.g. measurements) must be used to optimize λ. However, we are generally not concerned with the dynamics of the underlying generative model as λ is varied and instead will assume that ℙ(x|λ) is a steady-state distribution that can be approximated via sampling. In many cases, the dimension of the data distribution may be less than the dimension of the model distribution: dim(ψ)<dim(ℙ). In these cases, the extra dimensions of the model ℙ can be thought of as containing hidden (or latent) variables which may make the model more expressive and thereby help with the minimization process. Indeed, the success of so-called deep learning is partially attributed to the ability of large nonlinear latent spaces being able to represent complex functions and distributions [39].

### 2.2. Boltzmann machines

As an archetypal learning system, we will consider the generative graphical models called Boltzmann machines (BMs), which have been widely studied [36]. Briefly, a BM is a stochastic neural network with state $x\in \{0,1{\}}^{N}$, where N are the number of nodes in the network. These nodes form a graph where the vertices fluctuate between on (1) and off (0) states. Every state x of this graph is associated with a probability given by the distribution


(2.2) $$P(x)=\frac{1}{Z}e^{-E(x)}\;\;Z=\sum_{\begin{array}{c} x\in \{0,1{\}}^{N} \end{array}}e^{-E(x)}\;\;E(x)=\sum_{i>j}w_{ij}x_{i}x_{j}+\sum_{i}\theta_{i}x_{i}.$$

Here, the parameters, λ=(θ,w), are real-valued weights of the vertices and edges of the graph, respectively. The function E(x) is called the energy function. When a node i is on, its weight, $\theta_{i}$, is added to the energy. Similarly, only when both nodes i and j are jointly on does the edge connecting them, $w_{ij}$, contribute to the energy function. BMs are capable of probabilistic inference (e.g. computing conditional distributions) by holding a subset of their nodes constant.

Consider the nodes x divided into two groups u and v, such that (u,v)=x. The conditional distribution ℙ(u|v) can be computed by sampling the BM distribution with Markov chain Monte Carlo while not allowing v to vary; the variables v are said to be *clamped* and ℙ(u|v) is called the clamped distribution in contrast to the *free* distribution ℙ(u,v). In contexts where the same variables are being repeatedly clamped, perhaps to different environmental signals at different times, we say that v is the *visible* nodes while u is the *hidden* nodes which aid in representing complex distributions over v.

The parameters w and θ of a BM can also be learned via gradient descent on the relative entropy cost function


(2.3) $$\begin{array}{r} L(P,\psi )=D(\psi (v)||P(v))=\sum_{v}\psi (v)\;\log\frac{\psi (v)}{P(v)} \end{array},$$

where $P(v)=\sum_{u}P(u,v)$ is the marginal of ℙ(u,v) and the data are sampled from a distributed ψ(v). Note that the relative entropy is not symmetric: D(ψ∣∣P)≠D(P∣∣ψ). In the form of equation (2.3), we can consider ψ to be the ‘true’ distribution, while ℙ is an approximation; this corresponds to the relative entropy being an average over ψ: $D(\psi |P)=\langle \log\frac{1}{P}\rangle_{\psi }-\langle \log\frac{1}{\psi }\rangle_{\psi }$. This equation can be interpreted as the average excess information required to approximate ψ with ℙ. In order to allow BMs to model increasingly complex distributions, hidden units can be explicitly optimized via gradient descent on the relative entropy between the *clamped* distribution $\bar{P}=P(u|v)\;\psi \;(v)$ of the BM held to samples from ψ and the *free* distribution ℙ(u,v) of the BM


(2.4) $$D(\bar{P}||P)=\sum_{v,u}\bar{P}(u,v)\log\frac{\bar{P}(u,v)}{P(u,v)}\;\text{where}\;\bar{P}(u,v)=P(u|v)\;\psi \;(v).$$

Taking the gradient to $w_{ij}$ and $\theta_{i}$ results in the update rule which looks identical for both hidden and visible units


(2.5) $$\begin{array}{rlrl} & \frac{\text{d}w_{ij}}{\text{d}t}=\frac{\partial D(\bar{P}||P)}{\partial w_{ij}}=\epsilon (\langle x_{i}x_{j}\rangle_{\bar{P}}-\langle x_{i}x_{j}\rangle_{P}) & & \frac{\text{d}\theta_{i}}{\text{d}t}=\frac{\partial D(\bar{P}||P)}{\partial \theta_{i}}=\epsilon (\langle x_{i}\rangle_{\bar{P}}-\langle x_{i}\rangle_{P}). \end{array}$$

Here, $\langle \cdot \rangle_{\mathbb{P}}$ and $\langle \cdot \rangle_{\bar{P}}$ denote the expected value to the free and clamped distributions, respectively, and ϵ is the learning rate. Notice that the learning algorithm—gradient descent on the relative entropy—gives rise to dynamics for the parameters w and θ.

### 2.3. Chemical reaction networks

CRNs are a common model of well-mixed chemical environments, meaning that continuous spatial dynamics are neglected [40]. CRNs are a widely used modelling language for synthetic and systems biology and have been studied from many perspectives, including computer science, mathematics and statistical physics [27,31,40]. We denote a CRN (S,R,k) to be a set of species $S_{i}\in S$, a set of reactions R and rate constants k for each reaction. Species represent chemical entities, such as molecules. Reactions convert one multi-set of species to another: $I^{r}\overset{k_{r}}{\to }O^{r}$, where $I_{i}^{r}$ and $O_{i}^{r}$ are vectors denoting the number of species $S_{i}$ in the input and output of the reaction r, respectively. In the limit of a system with infinite volume but finite concentrations, a CRN defines deterministic dynamics of the species’ concentrations


(2.6) $$\frac{\text{d}[S_{i}]}{\text{d}t}=\sum_{r}M_{i}^{r}\eta_{r}([S])\;with\;\eta_{r}([S])=k_{r}\prod_{i}[S_{i}{]}^{I_{i}^{r}}.$$

Here, $M_{i}^{r}=O_{i}^{r}-I_{i}^{r}$ is the stoichiometric matrix and we will assume that all reactions occur according to mass action rates $\eta_{r}([S])$ unless explicitly noted otherwise.^1^ CRNs can also be considered stochastically in a finite volume V, in which case the dynamics of the probability that the species S will have counts s at time t is modelled using the chemical master equation


(2.7) $$\frac{\text{d}P(s,t)}{\text{d}t}=\sum_{r}P(s-M^{r},t)\rho_{r}(s-M^{r})-P(s,t)\rho_{r}(s)\;with\;\rho_{r}(s)=\frac{k_{r}}{V^{|I^{r}|-1}}\prod_{i}\frac{s_{i}!}{(s_{i}-I_{i}^{r})!}.$$

Here, all reactions occur with probability proportional to their mass-action propensity $\rho_{r}(s)$, which is equal^2^ to the mass-action rate $\eta_{r}([S])$ in the limit V→∞ while $s_{i}=V[S_{i}]$ with constant concentrations. We will often be interested in the stationary or steady-state distribution ${\mathbb{P}}^{*}(s)$ found by equating (2.7) to 0 or by simulating the CRN using the Gillespie algorithm until convergence [41]. It is worth noting that not all CRNs have unique or well-defined steady-state distributions—a technicality that will play a role in this article later.

### 2.4. Detailed balanced chemical reaction networks

Detailed balanced CRNs (dbCRNs) are a subclass of stochastic CRNs with the following properties:

— Each species $S_{i}\in S$ has a formal energy $G_{i}$.
— All reactions are reversible, meaning if $I\overset{k^{+}}{\to }O\in R$ then $O\overset{k^{-}}{\to }I\in R$.
— Reaction rates obey $\frac{k^{+}}{k^{-}}=e^{-\Delta G}$, where $\Delta G=\sum_{i}G_{i}(O_{i}-I_{i})$.

A dbCRN’s stationary distribution is an equilibrium distribution when there is no net energy flow or entropy production at a steady state. Detailed balanced CRNs may or may not be mass-conserving, meaning their equilibrium distribution can correspond to both the canonical or grand canonical ensembles of statistical physics. Regardless of the ensemble, all dbCRNs have a steady-state distribution with Poisson form written in terms of the free energy function G [42]


(2.8) $$\pi (s)=\frac{1}{Z}\prod_{i}\frac{e^{-G_{i}s_{i}}}{s_{i}!}=\frac{1}{Z}e^{-G(s)}\;\;Z=\sum_{s\in \Gamma_{s(0)}}e^{-G(s)}\;\;G(s)=\sum_{i}G_{i}(s)=\sum_{i}G_{i}s_{i}+\log s_{i}!.$$

Here, Z is called the partition function and $\Gamma_{s(0)}$ is called the reachability class that represents the set of all states reachable by any sequence of reactions in R from the initial condition s(0). In this article, we will emphasize that there are classes of dbCRNs with the same species and reactions but different rate constants reflecting different species’ energies. Let D=(S,R,k) be a detailed balanced CRN, then $D_{G}=\left(S,R,k_{G}\right)$ is the same set of species and reactions with the rate constants k changed to $k_{G}$ to be compatible with the new energies G. We note that $k_{G}$ is not necessarily unique, as the forward and backward rate constants for any single reaction can always be rescaled together by the same factor without breaking detailed balance, and thus, the rescaled dbCRN achieves the same equilibrium distribution.

## 3. Relation to prior work

### 3.1. Detailed balanced chemical reaction networks can model complex environments

Despite the canonical product Poisson form, specific dbCRNs are capable of representing complex distributions [34,35]. In recent work, we unify these observations and provide conditions under which a dbCRN can represent distributions that appear to be different from a product Poisson distribution [38]. Briefly, for a dbCRN to be far from product Poisson requires reactions and an initial condition, which together restrict the size of the reachability class relative to the full positive integer lattice. This fact is relevant because it provides an existence proof and suggests some design criteria for determining dbCRN reaction networks that may be capable of learning complex and far-from-Poisson distributions. In this work, we will show how these dbCRN models can be trained by coupling them with a non-detailed balanced CRN that implements the gradient descent algorithm.

### 3.2. Inference with detailed balanced chemical reaction networks

*Any* dbCRN is capable of probabilistic inference. First, we restate some definitions from [38]. Split the species in the dbCRN into disjoint sets of free and clamped species $(S^{F},S^{C})=S$:

— A *clamped* dbCRN $D_{G^{C}}=\left(S,R,k_{G^{C}}\right)$ is a dbCRN with its energies $G^{C}=G+\Delta$ where typically $\Delta_{i}\neq 0$ for any clamped species $S_{i}\in S^{C}$. The equilibrium distribution of this dbCRN is denoted $\pi_{C}$ and is given by equation (2.8) with energy vector $G^{C}$ replacing G.
— A *potentiated* dbCRN $D_{G}^{P}=\left(S\cup P,R^{P},k_{G}\right)$ is a dbCRN where each clamped species $S_{i}\in S^{C}$ is coupled to a potential species $P_{i}\in P$ such that whenever $S_{i}$ is created or destroyed by any reaction, its corresponding potential species is created or destroyed with it. The bulk concentration $\left[P_{i}\right]$ of potential species $P_{i}$ is held constant by the stochastic system being in contact with an infinite potential bath at equilibrium [43,44]. Therefore, every potentiated dbCRN is detailed balanced with equilibrium distribution $\pi (s|[P])=\pi^{P}(s)$ which depends on the potential species’ concentrations [P]. For example, the potentiated reactions of the one-reaction dbCRN with R={∅⇌xSi} are RP={∅⇌xSi+Pi}.

Previously, we showed that the following three notions of clamping are equivalent [38]:

1. *Sample clamping*. Collecting a biased sample of a dbCRN $D_{G}$’s dynamics such that the sampled mean $\langle s^{C}\rangle =\bar{c}$. The bias is achieved by running unbiased trajectories and rejecting those whose means deviate from the target in the appropriate limit.
2. *Energy clamping*. Varying the energies Δ in a clamped dbCRN $D_{G_{C}}$ such that $\langle s^{C}\rangle_{\pi_{C}}=\bar{c}$.
3. *Potential clamping*. Varying the potential species’ concentrations $\left[P_{i}\right]$ in a potentiated dbCRN $D_{G}^{P}$: $\Delta_{i}=G_{i}^{P}+\log[P_{i}]$ where $G_{i}^{P}$ is the energy of the potential species $P_{i}$.

In other words, conditioning on a dbCRN’s clamped species having a particular mean value, modulating the energies of a clamped dbCRN, and changing the concentrations of the potential species of a potentiated dbCRN are all equivalent forms of computing a conditional distribution $\pi (s^{F}|\langle s^{C}\rangle =\bar{c})$. These operations all are forms of probabilistic inference well suited for chemical systems and capable of highly complex computations when used on dbCRNs’ equilibrium distributions with highly restricted reachability classes [38]. In this work, our implementation of gradient descent will rely on coupling the dynamics of the potential species of a potentiated dbCRN with the computation of a conditional (clamped) distribution governed by those same potential species.

### 3.3. *In silico* learning for detailed balanced chemical reaction networks

In our previous work on chemical BMs [35], we showed that a version of the BM learning rule (2.5) also works for dbCRNs when viewed as a formal operation which can be performed *in silico*


(3.1) $$\frac{\text{d}G_{i}}{\text{d}t}=\frac{\partial D(\bar{\pi }||\pi )}{\partial G_{i}}=\epsilon (\langle s_{i}\rangle_{\bar{\pi }}-\langle s_{i}\rangle_{\pi }).$$

Naively, unlike the BM learning rule, equation (3.1) appears to only utilize the means of each species and not higher moments such as the second moments in equation (2.5). However, a detailed balanced construction called the *Edge-Species Chemical Boltzmann Machine* (ECBM) has been shown to also learn second moments [35]. We describe this model in electronic supplementary material, appendix C.

In this work, we will develop an autonomous CRN implementation of the learning rule (3.1) that works on any dbCRN, including networks with hidden species. To do this, we will make use of the potential clamping construction in the context of many vesicles (or proto-cells) interacting with a large environment, necessitating the use of an augmented hybrid CRN model that includes both deterministic and stochastic aspects.

## 4. Results

### 4.1. Overview

Our main result is a general CRN architecture that allows any potentiated dbCRN to autonomously learn a distribution from data (e.g. its environment). The key feature of this construction is a set of non-detailed balanced *potential clamping reactions* that modulate the potential species of the dbCRN to tune the underlying distribution. We organize our results as follows:

1. We describe a non-detailed balanced CRN, the potential clamping CRN, which forms the basic learning unit in our construction capable of computing conditional distributions when connected to a dbCRN and is a form of integral feedback control.
2. We generalize this construction to an ensemble of vesicles resulting in the ensemble potential clamping CRN. This step is necessary to guarantee a unique steady state for the entire construction.
3. We show how to use ensemble potential clamping CRNs to construct an autonomous learning CRN which is an online continuous time implementation of the BM learning algorithm (2.5) and is capable of engaging hidden units (e.g. latent variables represented as chemical species) to learn distributions.

We emphasize that our final learning CRN is an autonomous CRN that automatically learns from the environment, or in other words, we have completely rewritten a fundamental machine learning algorithm as a CRN. This demonstrates a mechanism by which a chemical system, such as a cell, can learn internal models of its environment. In the discussion, we comment on some possible biological scenarios where this behaviour may be advantageous and how we may find evidence of this behaviour in biological data.

### 4.2. Potential clamping chemical reaction network

In this section, we define the potential clamping CRN and describe its general behaviour. First, we consider a potentiated dbCRN, $D_{G}^{P}=\left(S\cup P,R_{P},k\right)$, as defined in the previous section. We will then add the following species and reactions to $D_{G}^{P}$ and construct a new potential clamping CRN. For each potential species, $P_{i}\in P$, add in target species $Q_{i}\in Q$ that later will be allowed to fluctuate according to some distribution ψ(q). We are agnostic about how ψ is generated—it could be equilibrium or non-equilibrium. Additionally, add the non-detailed balanced reactions, $T_{S,Q}^{P}$, that clamp the species S to the species Q using the potentials P


(4.1) $$T_{S,Q}^{P}=\left\{\begin{array}{c} P_{i}+Q_{i}\overset{\epsilon k_{i}^{Q}}{\to }Q_{i}\\ P_{i}+S_{i}\overset{\epsilon k_{i}^{S}}{\to }2P_{i}+S_{i} \end{array}\right\}.$$

Here, ϵ≪max⁡(k) act as a scaling parameter so the potential clamping reactions occur more slowly than any of the detailed balanced reactions in $R_{P}$ such that sub-network $D_{G}^{P}$ is at quasi-equilibrium relative to the chemical reaction network $T_{S,Q}^{P}$. Denote the potential clamping CRN as $C_{G}^{Q}=\left(S\cup P\cup Q,R_{P}\cup T_{S,Q}^{P},k\right)$. Intuitively, the behaviour of this CRN can be understood by considering the steady state behaviour of the potential species P in the deterministic limit


(4.2) $$\begin{array}{lrr} & 0=\frac{\text{d}[P_{i}]}{\text{d}t}=\epsilon [P_{i}]([S_{i}]k_{i}^{S}-[Q_{i}]k_{i}^{Q})\;⟹\;[P_{i}]=0\;\text{or}\;k_{i}^{S}[S_{i}]=k_{i}^{Q}[Q_{i}] & \end{array}$$

**Figure 1.**
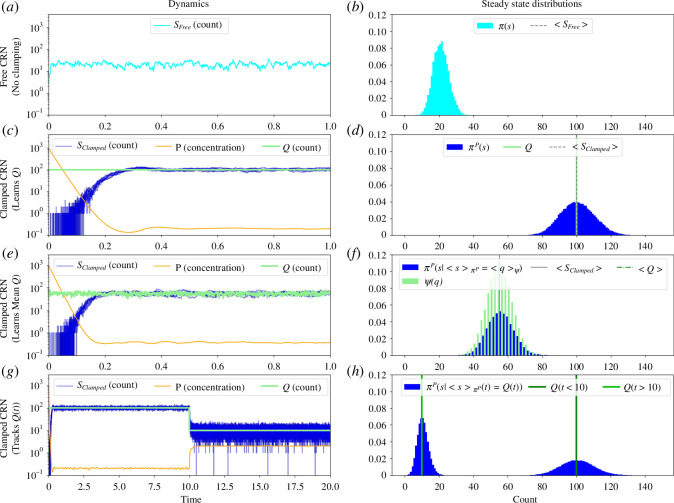
Dynamics and steady state distribution of potential clamping a single species S to Q using potential species P. (*a*) The free (unclamped) dynamics of a dbCRN ∅⇌xS. (*b*) Free steady state distribution of S. (*c*) Dynamics of S clamped to a constant value Q. (*d*) The mean clamped steady state distribution of S matches Q. (*e*) Dynamics S clamped to a quickly varying Q. (*f*) The mean of the clamped steady state distribution matches the mean of Q. (*g*) Dynamics of S clamped to a slowly varying Q exhibits reference tracking. (*h*) The clamped distribution of S has a mode matching each value of Q. Notice that in all these different cases, the potential clamping reactions are very effective at matching S to Q.

This means either $\left[P_{i}\right]$ is 0 or $S_{i}$ equals $Q_{i}$ (up to a proportionality constant). The latter case corresponds to $S_{i}$ tracking the value $Q_{i}$. This is illustrated in figure 1, which shows the behaviour of the potential clamping reactions coupled to a birth–death process ∅⇌xS+P. These dynamics can also be interpreted as a form of feedback control where the reactions $T_{S,Q}^{P}$ are the controller which uses the potential species P to modulate the dbCRN (plant) species S to match the values of the target species Q. In electronic supplementary material, appendix F, we make this intuition rigorous by proving that the potential clamping reactions are a form of integral feedback. Additionally, we comment that the potential clamping CRN can also be thought of as computing a kind of conditional distribution [38], π(s|⟨s⟩=Q), where the potential clamping reactions modulate the mean value of S to the mean value of Q.

Notice that $C_{G}^{Q}$ is no longer detailed balanced, and instead of an equilibrium distribution, it may have a non-equilibrium steady state, ℙ(s,q,p) which may be hard to analyse. Indeed, in electronic supplementary material, equation (14), we provide an example illustrating that this steady-state distribution may not be unique. The intuition behind the existence of multiple steady states is the possibility for extinction (meaning 0 counts) of the $P_{i}$ species and the $S_{i}$ species simultaneously, resulting in the dynamics of the CRN halting completely due to the fact that $P_{i}$ and $S_{i}$ are inputs to every reaction in the system by construction. The conditions for extinction events have been characterized in the context of stochastic CRNs [45], and it may be possible to develop learning systems that are robust to these effects. However, this potentially pathological behaviour makes a fully stochastic version of our system intractable as a general model of learning and motivates the use of an ensemble of potential clamping CRNs.

### 4.3. Ensemble potential clamping chemical reaction networks

**Figure 2.**
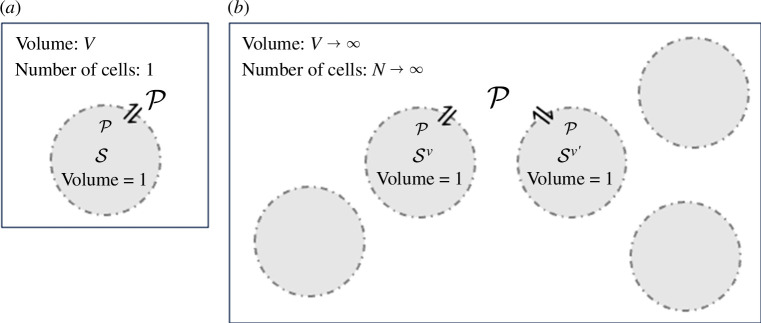
(*a*) A cartoon illustration of a single unit volume vesicle in a large but finite volume V with internal species S and potential species P. (*b*) A cartoon illustration of the vesicle ensemble where N→∞ vesicles with distinct internal species $S^{v}$ share potential species P in an infinite volume.

In this section, we define the ensemble potential clamping CRN, which can be thought of as a stochastic–deterministic hybrid model of the potential clamping CRN. This step is necessary to remedy the pathological behaviour of the fully stochastic potential clamping CRN. We consider the deterministic limit of an ensemble of N identically and independently distributed vesicles (with unit volumes for simplicity) in a large volume V as illustrated in figure 2. Each vesicle v contains its copy of the same potentiated dbCRN consisting of species $S^{v}$ that cannot diffuse through the vesicle’s membrane and reactions $R_{P}^{v}$. However, all vesicles share the same potential species P that rapidly diffuse through their membranes. Crucially, any species $S_{i}^{v}$ and $S_{i}^{v^{\prime }}$ are coupled to the same potential species $P_{i}$. Intuitively, an ensemble of independent vesicles in a large volume will have a very low chance of many simultaneous extinction events. As we prove in electronic supplementary material, appendix E, this entire system can be reduced to a non-mass-action CRN governing the potential species


(4.3) $$P_{i}+Q_{i}\overset{\epsilon k_{i}^{Q}}{\to }Q_{i}\;\;\;P_{i}+S_{i}\overset{\rho_{i}^{S}(s,p,\pi^{P})}{\to }2P_{i}+S_{i}\;\;\;\rho_{i}^{S}(s,p,\pi^{P})=\epsilon k_{i}^{S}p_{i}\nu \langle s_{i}\rangle_{\pi^{P}}.$$

Here, the first reaction uses the standard mass-action propensity, ν=N/V is the vesicle concentration, and $\langle s_{i}\rangle_{\pi^{P}}$ is the expected value of $S_{i}$ to the quasi-equilibrium distribution $\pi^{P}$. Due to the inclusion of expected values, it is unclear how to simulate this CRN stochastically. However, as we show in electronic supplementary material, appendix E, this CRN can be interpreted as a hybrid deterministic–stochastic system in the limit: ϵ→0, V→∞, N→∞ and $p_{i}\to \infty$ such that $[P_{i}]=\frac{p_{i}}{V}$ and $\nu =\frac{N}{V}$. This limit results in the ODEs


(4.4) $$\begin{array}{lrr} & \frac{\text{d}[P_{i}]}{\text{d}t}=\epsilon \left(k_{i}^{S}[P_{i}]\nu \langle s_{i}\rangle_{\pi^{P}}-k_{i}^{Q}[P_{i}][Q_{i}]\right). & \end{array}$$

This ODE is equivalent to the moment learning rule as seen by rewriting equation (3.1) using the explicit form of the free energy change $\Delta_{i}=G_{i}^{P}+\log\;P_{i}$


(4.5) ddtΔi=ddt(GiP+log⁡Pi)=1Pid[Pi]dt(6)=ϵ(kiSν⟨si⟩πP−kiQ[Qi]).

The constant ϵ is analogous to a learning rate. The extra constants $k_{i}^{S}$, $k_{i}^{Q}$ and ν can be interpreted as scale factors controlling the relative abundances of the target species Q, the concentrations of vesicles ν and the counts of S. In other words, at steady state $S_{i}$ is clamped to a mean value proportional to $Q_{i}$ or $\left\langle Q_{i}\right\rangle$ as illustrated in figure 1


(4.6) $$\begin{array}{r} \frac{\text{d}[P_{i}{]}_{ss}}{\text{d}t}=0\;⟹\;\langle s_{i}\rangle_{\pi^{P}}=\left\{\begin{array}{cc} \alpha_{i}[Q_{i}] & Q_{i}\text{ is constant}\\ \alpha_{i}\langle Q_{i}\rangle & Q_{i}\text{ varies quickly} \end{array}\right\}\;\text{with}\;\alpha_{i}=\frac{k_{i}^{Q}}{k_{i}^{S}\nu } \end{array}.$$

The dynamics of this system can also be solved assuming [Q] is constant


(4.7) $$\begin{array}{rl} \frac{\text{d}[P_{i}](t)}{\left[P_{i}](t\right)} & =\epsilon (k_{i}^{S}\nu \langle s_{i}\rangle_{\pi^{P}}-k_{i}^{Q}\langle q_{i}(t)\rangle_{\psi })\text{d}t\\ \;⟹\;[P_{i}](t) & =Ce^{\epsilon (k_{i}^{S}\nu \langle s_{i}\rangle_{\pi^{P}}-k_{i}^{Q}[Q_{i}](t))} \end{array}$$

where C is a constant of integration. In words, the CRN $C_{G}^{Q}$ will vary $\left[P_{i}\right]$, which is equivalent to varying the energy of $S_{i}$ via the chemical potential $\mu_{i}$: $\Delta_{i}=\mu_{i}=G_{i}^{P}+\log[P_{i}]$. Furthermore, if the rates ϵ are small enough and the correct initial condition is chosen, this system may reach a fixed point $[P_{i}{]}_{ss}$ such that equation (4.6) is satisfied—meaning that the mean of $S_{i}$ is equal to the mean of $Q_{i}$ multiplied by the scaling factor $\alpha_{i}$. The dynamics also illustrate that clamping will be fast with exponential convergence to the correct value provided there is no error in estimating the expected value $\langle s\rangle_{\pi^{P}}$. However, as these distributions become noisier (meaning not enough time-scale separation), the exponential will amplify fluctuations and learning becomes a biased random walk. Importantly, some level of fluctuations in the dynamics may help convergence to an optimal solution, just as stochastic gradient descent using mini-batches frequently converges better than deterministic gradient descent implementations by avoiding spurious local minima. Figure 1 illustrates the dynamics of the potential clamping reactions when applied to a birth–death process of S which is shown unclamped in panel *a*. In panel *c*, the target species Q is held constant and S is clamped to its value [Q]. In panel *e*, Q fluctuates quickly and S is clamped to $\langle Q\rangle_{\psi }$. Finally, in panel *g*, the Q varies slowly and the potential clamping CRN can be viewed as a form of integral feedback control which causes S to track the reference signal $\left[Q_{i}](t\right)$.

### 4.4. Autonomous learning chemical reaction networks with hidden units

In this section, we show how to construct an autonomous learning CRN capable of dynamically adjusting potential species so that an internally potentiated dbCRN matches an environmental distribution. This construction is an online continuous-time version of the BM learning rule. The final CRN has three components: (i) species and reactions that sample the clamped distribution, (ii) species and reactions that sample the free distribution, and (iii) species and reactions coupling these two distributions, such that they implement the learning rule (2.5).

*Computing the clamped distribution*. Let the environmental distribution ψ have visible species $Q^{V}∼\psi$. The clamped species are split into visible and hidden sets $\bar{S}=({\bar{S}}^{U},{\bar{S}}^{V})$ and corresponding potential species $\bar{P}=(P^{U},{\bar{P}}^{V})$ interact as a potentiated dbCRN via the reactions $R_{\bar{P}}^{\bar{S}}$. Furthermore, the visible species ${\bar{S}}^{V}$ are explicitly clamped to the environmental species $Q^{V}$ via the potential clamping reactions $T_{{\bar{S}}^{V},Q^{V}}^{{\bar{P}}^{V}}$. If Q fluctuates very quickly, $\langle s^{V}\rangle =\left\langle q^{V}\right\rangle$. On the other hand, if Q fluctuates much more slowly than the potential clamping reaction $s^{V}$ will track $q^{V}$. This latter case implements the clamped distribution $\bar{\pi }({\bar{s}}^{U},{\bar{s}}^{V})=\pi ({\bar{s}}^{U}|{\bar{s}}^{V})\;\psi \;(q^{V})$.

*Computing the free distribution*. The free species $S=\left(S^{U},S^{V}\right)$ and corresponding potential species $P=\left(P^{U},P^{V}\right)$ interact as a potentiated dbCRN via the reactions $R_{P}^{S}$ which are identical to the reactions $R_{\bar{P}}^{\bar{S}}$ but use the free species as opposed to their clamped counterparts. These reactions compute the free distribution, $\pi (s^{U},s^{V})$. Note that the hidden potential species $P^{U}$ are used in both $R_{P}^{S}$ and $R_{\bar{P}}^{\bar{S}}$ analogously to the way that the free and clamped distributions of a BM share the same energy function parameters.

*Computing the learning rule*. The free and clamped species are coupled by a learning rule that tunes the potential species P by clamping the free species to the clamped species. This is implemented by a second set of potential clamping reactions $T_{S,\bar{S}}^{P}$. Analogously to equation (4.5), these learning reactions induce dynamics on the potential species


(4.8) $$\frac{1}{\left[P_{i}\right]}\frac{\text{d}[P_{i}]}{\text{d}t}=\epsilon_{learn}\left(\langle s_{i}\rangle_{\pi }-\langle {\bar{s}}_{i}\rangle_{\bar{\pi }}\right),$$

where we have let $k_{i}^{S}\nu =k_{i}^{\bar{S}}=1$ to highlight the equivalence between these dynamics and the BM learning rule (2.5). We emphasize that these reactions are responsible for engaging hidden units because they clamp all the free species to all the clamped species (not just the visible ones), analogous to the way the moments of both visible and hidden units in a BM are used to update the parameters of the model.

*The autonomous learning CRN*. By combining the modules described above, we arrive at a completely autonomous learning CRN


(4.9) $$L_{G}^{Q}=(Q\cup \bar{S}\cup S\cup \bar{P}\cup P,R_{\bar{P},P}^{\bar{S}}\cup R_{P}^{S}\cup T_{\bar{S},Q}^{\bar{P}}\cup T_{S,\bar{S}}^{P},k).$$

**Figure 3.**
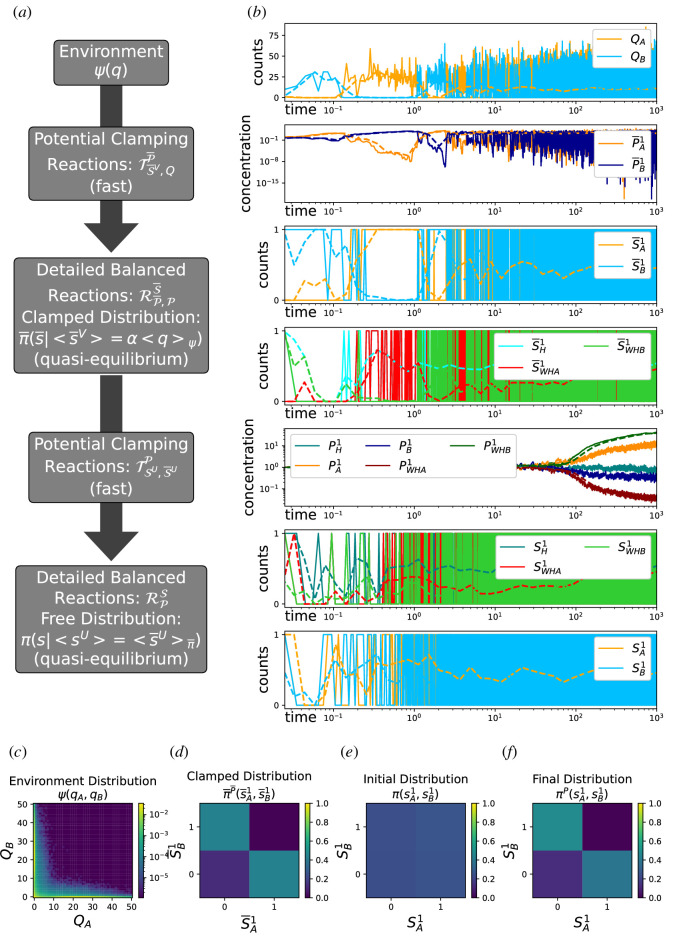
(*a*) The learning architecture coupling dbCRNs and the environment together with potential clamping reactions. Arrows point from the target species to the clamped species. (*b*) Trajectories from this architecture applied to learn an XOR distribution with a three-node chemical Boltzmann machine (CBM) from a bistable genetic toggle switch. Note that the X-axis (time) is logarithmic. Solid lines show trajectories for a single training iteration. Dashed lines denote the time-binned means of similarly coloured species. (*c*) The steady state distribution of the bistable toggle switch. (*d*) The distribution obtained from clamping to the environment. (*e*) The initial distribution of the CBM before training. (*f*) The final distribution of the CBM after training.

This CRN uses two sets of potential clamping reactions simultaneously to clamp the clamped species to the environment and then clamp the free species to the clamped species as illustrated in figure 3*a*. Although seemingly complicated, $L_{G}^{Q}$ is implementing a version of the moment learning algorithm of BMs as a continuous-time online process in the sense that the free and clamped distributions are being continuously sampled and in turn drive a continuous update of the potential species.

We illustrate an example of $L_{G}^{Q}$’s dynamics in figure 3*b*. The visible environment has two visible species $Q_{A}$ and $Q_{B}$ fluctuating according to an XOR distribution generated by a stochastic bistable toggle switch [46] (figure 3*c*). Any dbCRN could, in principle, be used for the clamped and free dbCRN models. We have chosen to use two three-node chemical Boltzmann machines (CBMs), described in electronic supplementary material, appendix C, as the clamped and free dbCRN models because they are known to be able to represent complex distributions using hidden units. The *clamped* CBM has two visible nodes represented by pairs of binary species ${\bar{S}}_{A}^{\;\beta }$ and ${\bar{S}}_{B}^{\;\beta }$ and a single hidden node represented by the species ${\bar{S}}_{H}^{\;\beta }$. Edges that connect the hidden node to each visible are represented by the species ${\bar{S}}_{W^{HA}}^{\;\beta }$ and ${\bar{S}}_{W^{HB}}^{\;\beta }$. Here, β∈{0,1} are used to denote the *off* and *on* forms of each node. The species ${\bar{S}}_{A}^{1}$ and ${\bar{S}}_{B}^{1}$ are clamped to the environmental species $Q_{A}$ and $Q_{B}$, respectively, via the potential clamping reactions $T_{\bar{S},Q}^{\bar{P}}$ which modulate the species ${\bar{P}}_{A}$ and ${\bar{P}}_{B}$ (figure 3*d*). The *free* CBM has the same topology as the *clamped* CBM: nodes are represented by species $S_{A}^{\beta }$, $S_{B}^{\beta }$ and $S_{H}^{\beta }$ with edges $S_{W^{HA}}^{\beta }$ and $S_{W^{HB}}^{\beta }$. The free CBM is clamped to the clamped CBM via the potential clamping reactions $T_{S,\bar{S}}^{P}$ which modulate the species $P_{A}$, $P_{B}$, $P_{H}$, $P_{W^{HA}}$ and $P_{W^{HB}}$. This second set of potential clamping reactions allows the free CBM to learn from the distribution of the clamped CBM, causing it to begin as a uniform distribution (figure 3*e*) and, by the end of the training, produce an XOR distribution (figure 3*f*). We emphasize that there are no direct interactions between the visible units of these dbCRNs, $S_{A}\;\;and\;\;S_{B}$, which means that to learn an XOR distribution, they must be engaging the hidden species $S_{H}$ and potential species $P_{W^{HA}},\;\;and\;\;P_{W^{HB}}$. We comment that the learned distributions are binary (as opposed to having support over larger portions of the integer lattice) because the CBM model we chose to train exactly implements a BM and, therefore, is restricted to binary representations. The factor α was used to rescale these distributions appropriately. We describe the learning system simulated in figure 3*b* in detail in electronic supplementary material, appendix G. Next, we comment that this construction appears quite large, particularly in the number of reactants required in each reaction. However, it is theoretically possible to closely approximate this high-order behaviour with bi-molecular reactions and in some cases may even be possible to do so relatively compactly [47].

This construction is quite general and can, in theory, be applied to any detailed balanced CRN, not just the CBM construction used in the simulation. We provide a detailed illustrative example of this construction applied to a simple architecture in electronic supplementary material, appendix B. Given a free potentiated dbCRN with $n_{S}$ non-potential species, $n_{R}$ reactions, $n_{P}$ potential species and $n_{Q}$ visible environmental species, the full learning CRN requires $n_{S}$ additional species, $n_{Q}$ additional potential species and $2*n_{p}+2*n_{Q}+n_{R}$ additional reactions to implement two sets of potential clamping reactions and the clamped dbCRN, making the complexity of this linear in the size of the free dbCRN plus the number of visible environmental species. Finally, although in this situation learning is active constantly, it is not hard to augment the construction so learning can be turned on and off, for example by setting ϵ=0 or having the potential clamping reactions catalysed by a species which can be controlled like a switch. We emphasize that this construction is a fully autonomous CRN capable of adapting diverse internal models to arbitrary environmental distributions.

## 5. Discussion

We have presented a general chemical system capable of converting any potentiated dbCRN into an autonomous learning system. Underlying this construction is a set of potential clamping reactions that continuously modulate the chemical potentials of the species in the dbCRN. We have shown that these reactions implement a CRN version of gradient descent on the relative entropy which, intriguingly, also takes the form of a feedback control circuit. Unlike previous chemical implementations of learning algorithms [19,35,38,48–52], our construction continuously and dynamically changes its parameters (the chemical potentials) in real time without external intervention. We further showed that the potential clamping reactions can be used to implement a continuous-time online version of the classic BM gradient descent learning algorithm, where one set of potential clamping reactions copies environmental variables into a clamped dbCRN and the second set of potential clamping reactions minimizes the relative entropy between the clamped dbCRN and a free dbCRN. This process allows for the free dbCRN to minimize its relative entropy with the environment, utilizing hidden species to increase the complexity of the distributions it can represent. We emphasize that, although this paper focuses on a chemical implementation of BMs, this construction can be applied to any dbCRN coupled with potential learning reactions. Finally, the concrete nature of our implementations allowed us to unambiguously analyse the thermodynamic costs of potential clamping and learning, which we do in electronic supplementary material, appendix H to show that there is a clear accuracy/dissipation trade-off in this model. This basic analysis adds to a growing body of literature addressing the fundamental physics of machine learning [48,53–55].

One particularly novel aspect of our construction is its use of an ensemble of stochastic vesicles. We note that this choice may not be strictly necessary as a hybrid deterministic–stochastic CRN model with a large separation of time scales can give similar behaviour [56]. However, using an ensemble of CRN-vesicles has a number of potential applications. First, such a system could be implemented in a laboratory using droplet-based technologies with potential species diffusing between droplets [57]. Second, the many-vesicle construction may also inspire novel machine-learning algorithms. Although our current *in silico* implementation is reliant on conventional chemical reaction network simulators and therefore involves minimal parallelism, it may be possible to exploit the ensemble-of-vesicles interpretation of this model to develop highly parallel *in silico* machine learning architectures based on chemical reaction network models.

Finally, we wish to comment on the importance of our results to understanding diverse biological systems. Our construction suggests that any multi-compartment system that communicates between compartments via diffusive molecules could potentially be performing an algorithm similar to what we have described in this article. Quorum sensing molecules such as *N*-Acyl homoserine lactones (AHLs) in bacteria [58], morphogens in eukaryotic development [59] and immune system responses modulated by cytokines [60] are three biological examples where diffusive molecules may be acting as adaptive global potentials biasing groups of cells to perform specific functions. Additionally, as we mentioned above, our construction is not necessarily reliant on multiple vesicles and, instead, could be reliant on the separation of time scales with a quickly equilibrating dbCRN modulating itself via slowly changing potential species. The epigenetic code and corresponding chromatin structure may be an example of such a system. Transcription factor binding and covalent histone and DNA modifications can occur relatively quickly compared with the time it takes for transcription, translation and maturation of new chromatin-modifying enzymes. In this sense, chromatin-binding proteins could be analogous to potential species with the chromatin structure analogous to the dbCRN. In electronic supplementary material, appendix I, we describe a biochemical network inspired by chromatin looping, which is capable of tuning its behaviour using the potential clamping reactions. Importantly, we note that this example is considerably more simple, involving fewer species and reactions, than a CBM. However, it is unclear if it can represent a complex of distributions. Obviously, biology is unlikely to exactly implement the learning constructions described in this article, but we posit that understanding biological computation from a machine-learning-inspired lens may prove fruitful.

## 6. Methods

All numerical simulations of chemical reaction networks were carried out with the Bioscrape Python package [61] using a hybrid deterministic–stochastic approach. Typically N=10 vesicles containing independent, detailed balanced chemical reaction networks were simulated via the Gillespie algorithm. Simultaneously, bioscrape rules were used to numerically integrate the dynamics of the potential species every dt. This results in a coupled deterministic–stochastic hybrid simulation that is accurate for sufficiently small dt. A value of dt=0.001 was determined to be sufficiently accurate provided the smallest rate in the system $\epsilon_{learn}\geq 0.01$ by comparing increasingly smaller values of dt and determining that the numerical output had converged. Steady-state distributions were found by simulating all systems until the distribution converged.

## Data Availability

The code used to generate the figures in this work is available at: [62]. Supplementary material is available online [63].

## Footnotes

^1^
For convenience, we will specify concentrations, volume and time in units such that a concentration of 1 indicates 1 molecule in a volume of 1 and a bimolecular reaction with both distinct reactants at concentration 1 will take place at rate 1.

^2^
Physically, rate constants in deterministic and stochastic equations have different meanings (due to being expressed with different units): the deterministic equations are volume-independent, while the values in the stochastic context must depend on volume (and can be related to the deterministic values given a choice of volume).
